# Improvement in Hydriding and Dehydriding Features of Mg–TaF_5_–VCl_3_ Alloy by Adding Ni and x wt% MgH_2_ (x = 1, 5, and 10) Together with TaF_5_ and VCl_3_

**DOI:** 10.3390/mi12101194

**Published:** 2021-09-30

**Authors:** Young-Jun Kwak, Myoung-Youp Song

**Affiliations:** Division of Advanced Materials Engineering, Jeonbuk National University, 567 Baekje-daero Deokjin-gu, Jeonju 54896, Korea; twistking18@nate.com

**Keywords:** hydrogen-storage materials, magnesium, hydriding and dehydriding rates, cycling performance, microstructure, addition of TaF_5_, VCl_3_, Ni, and MgH_2_

## Abstract

In our previous work, TaF_5_ and VCl_3_ were added to Mg, leading to the preparation of samples with good hydriding and dehydriding properties. In this work, Ni was added together with TaF_5_ and VCl_3_ to increase the reaction rates with hydrogen and the hydrogen-storage capacity of Mg. The addition of Ni together with TaF_5_ and VCl_3_ improved the hydriding and dehydriding properties of the TaF_5_ and VCl_3_-added Mg. MgH_2_ was also added with Ni, TaF_5_, and VCl_3_ and Mg-x wt% MgH_2_-1.25 wt% Ni-1.25 wt% TaF_5_-1.25 wt% VCl_3_ (x = 0, 1, 5, and 10) were prepared by reactive mechanical milling. The addition of MgH_2_ decreased the particle size, lowered the temperature at which hydrogen begins to release rapidly, and increased the hydriding and dehydriding rates for the first 5 min. Adding 1 and 5 wt% MgH_2_ increased the quantity of hydrogen absorbed for 60 min, H_a_ (60 min), and the quantity of hydrogen released for 60 min, H_d_ (60 min). The addition of MgH_2_ improved the hydriding–dehydriding cycling performance. Among the samples, the sample with x = 5 had the highest hydriding and dehydriding rates for the first 5 min and the best cycling performance, with an effective hydrogen-storage capacity of 6.65 wt%.

## 1. Introduction

Metal hydride storage has advantages over pressure storage and cryogenic storage, as metal hydrides have higher volumetric capacity and metal hydride storage is safer due to the low pressures involved in hydrogen uptake and release [1].

To increase the hydrogen uptake and release rates of magnesium, magnesium (Mg), or magnesium hydride (MgH_2_) was alloyed [2,3,4,5,6,7,8,9,10] with Ni and Y [11], V [12], and Nb [13]. In addition, Mg or MgH_2_ was mixed with compounds such as LaNi_5_ [14], FeTi and FeTiMn [15], Nb_2_O_5_ [16], and La_2_O_3_ [17]. Mg-containing compounds [18,19,20,21,22] such as La_2_Mg_16_Ni [23], LaMg_12_ and La_2_Mg_17_ [24], Mg_3_Mm (Mm: misch-metal) [25], and Mg_17_Al_12_ [26] were synthesized for the same purposes.

Malka et al. [27] reported that certain metal halide additives, especially ZrF_4_ and NbF_5_ halides, could significantly influence the sorption properties of MgH_2_. They reported that the presence of the F anion, which weakened Mg–H bonding, led to the formation of MgF_2_ and provided an electron-rich center to trap transition metal atoms. They showed that NbF_5_, TaF_5_, and particularly TiCl_3_, took part in the disproportionation reactions that created a significant number of structural defects. Kumar et al. [28] reported that VCl_3_ was reduced to metallic vanadium during ball milling along with MgH_2_, and this in situ-formed metallic vanadium doped over the MgH_2_ surface and showed an excellent catalytic effect on hydrogenation–dehydrogenation of the Mg-MgH_2_ system. They also reported that a microstructural analysis showed an excellent grain refinement property of VCl_3_ which reduced the crystallite size of MgH_2_. Liang et al. [13] improved the hydrogen-storage properties of Mg by adding transition metals such as Ti, V, Mn, Fe, and Ni. In order to find a new route toward improving hydrogen sorption kinetics of Mg nanoparticles, Liu et al. [29] coprecipitated a Mg–Ni nanocomposite from a homogeneous tetrahydrofuran solution containing anhydrous MgCl_2_, NiCl_2_, and lithium naphthalide as the reducing agent.

In the present work, we tried to improve the hydriding and dehydriding properties of Mg by adding halides, transition metal, and metal hydride by preparing alloys via reactive mechanical milling. Selected were TaF_5_ and VCl_3_ as halides, Ni as a metal, and MgH_2_ as a metal hydride. Reactive mechanical milling is milling in an atmosphere in which a reaction can occur during that milling. In the present work, milling was performed in a hydrogen atmosphere. In the hydrogen atmosphere, Mg hydride can form during milling through a reaction of Mg with hydrogen.

In our previous work [30], TaF_5_ and VCl_3_ were added to Mg. In this work, Ni was added together with TaF_5_ and VCl_3_; Ni, TaF_5_, and VCl_3_ were added to Mg at the same time. MgH_2_ was also added to Mg together with Ni, TaF_5_, and VCl_3_ at the same time, and Mg-x wt% MgH_2_-1.25 wt% Ni-1.25 wt% TaF_5_-1.25 wt% VCl_3_ (x = 0, 1, 5, and 10) were prepared by reactive mechanical milling. We designated these samples as Mg-xMgH_2_-1.25Ni-1.25TaF_5_-1.25VCl_3_ (x = 0, 1, 5, and 10). The hydriding and dehydriding properties of the prepared samples were then examined. It is thought that the materials developed in our work can be used for motive power fuel and portable appliances as mobile applications, transport and distribution as semi-mobile applications, and industrial off-peak power H_2_-generation, hydrogen-purifying systems, and heat pumps as stationary applications.

## 2. Materials and Methods

Pure Mg powder (particle size 74–149 μm, purity 99.6%, Alfa Aesar), Ni (APS 2.2–3.0 μm, purity 99.9% metal basis, C typically < 0.1%, Alfa Aesar, Haverhill, MA, USA), TaF_5_ (Tantalum (V) fluoride, purity 98%, Aldrich, St. Louis, MI, USA), VCl_3_ (Vanadium (III) chloride, purity 97%, Aldrich), and pure MgH_2_ powder (hydrogen storage grade, Aldrich) were used as starting materials.

The compositions of mixtures for reactive mechanical grinding were 96.25 wt% Mg + 1.25 wt% Ni + 1.25 wt% TaF_5_ + 1.25 wt% VCl_3_ and (96.25-x) Mg + x wt% MgH_2_ + 1.25 wt% Ni + 1.25 wt% TaF_5_ + 1.25 wt% VCl_3_ (x = 0, 1, 5, and 10). A planetary ball mill (Planetary Mono Mill; Pulverisette 6, Fritsch, Kastl, Germany) with a mill container of 250 mL in volume was used for reactive mechanical grinding. The sample (total weight = 8 g) to ball weight (105 hardened steel balls, total weight = 360 g) ratio was 1:45. All sample handling was performed in an Ar atmosphere. The disc revolution speed was 250 rpm. Reactive mechanical milling was performed in high purity hydrogen gas (≈12 bar) for 6 h by repeating the 20 min cycle of 15 min milling and 5 min rest. Hydrogen was refilled every two hours.

The absorbed and released hydrogen quantity were measured as a function of time by the volumetric method using a Sieverts’ type hydrogen uptake and release apparatus that was previously described [31]. The hydrogen pressure in the sample-containing reactor was maintained to be nearly constant (under 12 bar for the hydrogen uptake reaction and under 1.0 bar for the hydrogen release reaction) using a back-pressure regulator. The back-pressure regulator enables an appropriate amount of hydrogen to be taken from the standard reservoir (with a known volume) and dosed to the reactor during the hydrogen uptake reaction and an appropriate amount of hydrogen to be removed from the reactor to the standard reservoir during the hydrogen release reaction. From the temperature of the standard reservoir and the variation in hydrogen pressure in the standard reservoir (with a known volume) as a function of time, the variation in the absorbed or released hydrogen quantity was calculated as a function of time. The quantity of the samples used to measure the amount of absorbed or released hydrogen as time passed was 0.5 g. The standard deviations of the amount of absorbed and released hydrogen were ±0.07 wt% H.

Samples after reactive mechanical milling were characterized via X-ray diffraction (XRD) with Cu Kα radiation at a scan speed of 4°/min, using a Rigaku D/MAX 2500 powder diffractometer. The MDI JADE 5.0 program was used to analyze the XRD patterns. Scanning electron microscope (SEM) micrographs of the powders were obtained using a JSM-5900 SEM operated at 20 kV. Particle size distributions of the samples after reactive mechanical milling were analyzed by dynamic light scattering in a particle size analyzer (PSA, ASAP2010 Micrometrics, Norcross, GA, USA).

## 3. Results

The quantities of absorbed and released hydrogen, H_a_ and H_d_, respectively, were defined using sample weight as a criterion. H_a_ and H_d_ were expressed in the unit of wt% H. The quantities of hydrogen absorbed and released from the start for x min are expressed by H_a_ (x min) and H_d_ (x min), respectively. The hydriding rate for the first 5 min (wt% H/min) was calculated by dividing H_a_ (5 min) by 5 and the dehydriding rate for the first 5 min (wt% H/min) was calculated by dividing H_d_ (5 min) by 5.

**Figure 1:**
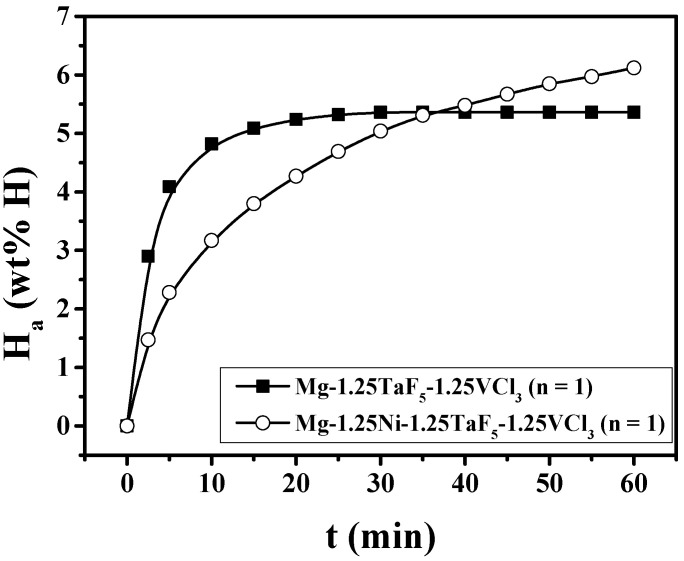
H_a_ vs. t curves at 593 K under 12 bar H_2_ at n = 1 for Mg-1.25TaF_5_-1.25VCl_3_ and Mg-1.25Ni-1.25TaF_5_-1.25VCl_3_.

Figure 1 shows the H_a_ vs. time t curves at 593 K under 12 bar H_2_ at the number of cycles, n, of one (n = 1) for Mg-1.25TaF_5_-1.25VCl_3_ and Mg-1.25Ni-1.25TaF_5_-1.25VCl_3_. Mg-1.25Ni-1.25TaF_5_-1.25VCl_3_ has a lower hydriding rate for the first 5 min, but a larger quantity of hydrogen absorbed for 60 min than Mg-1.25TaF_5_-1.25VCl_3_. Mg-1.25Ni-1.25TaF_5_-1.25VCl_3_ absorbs 2.28 wt% H for 5 min, 3.17 wt% H for 10 min, 5.04 wt% H for 30 min, and 6.12 wt% H for 60 min.

**Figure 2:**
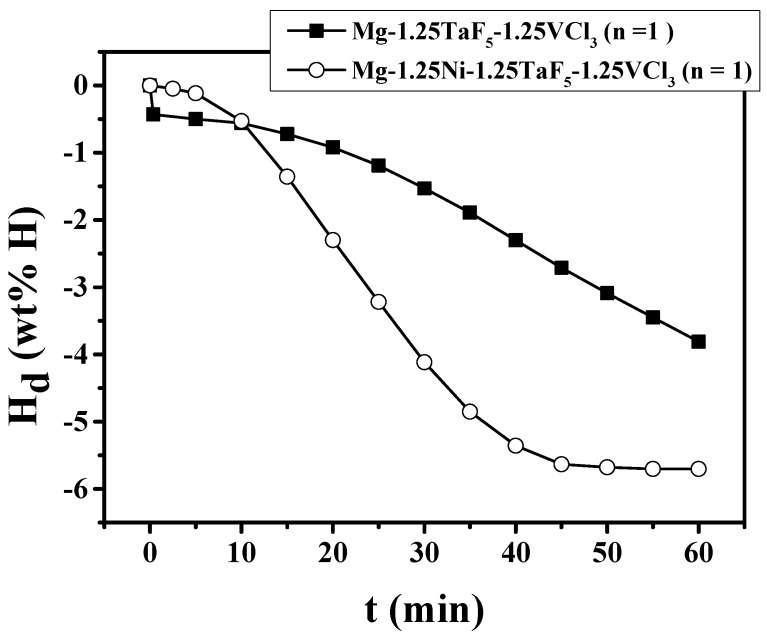
H_d_ vs. t curves at 593 K under 1.0 bar H_2_ at n = 1 for Mg-1.25TaF_5_-1.25VCl_3_ and Mg-1.25Ni-1.25TaF_5_-1.25VCl_3_.

The H_d_ vs. time t curves at 593 K under 1.0 bar H_2_ at n = 1 for Mg-1.25TaF_5_-1.25VCl_3_ and Mg-1.25Ni-1.25TaF_5_-1.25VCl_3_ are shown in Figure 2. Mg-1.25TaF_5_-1.25VCl_3_ shows a maximum dehydriding rate after about 20 min. On the other hand, Mg-1.25Ni-1.25TaF_5_-1.25VCl_3_ shows a maximum dehydriding rate after about 15 min. Mg-1.25Ni-1.25TaF_5_-1.25VCl_3_ has a higher maximum dehydriding rate and a larger quantity of hydrogen released for 60 min than Mg-1.25TaF_5_-1.25VCl_3_. Mg-1.25Ni-1.25TaF_5_-1.25VCl_3_ releases 0.11 wt% H for 5 min, 0.53 wt% H for 10 min, 4.11 wt% H for 30 min, and 5.70 wt% H for 60 min.

Figure 1 shows that the addition of Ni together with TaF_5_ and VCl_3_ increases the quantity of hydrogen absorbed for 60 min of the TaF_5_ and VCl_3_-added Mg. Figure 2 shows that the addition of Ni together with TaF_5_ and VCl_3_ increases the maximum dehydriding rate and the quantity of hydrogen released for 60 min of the TaF_5_ and VCl_3_-added Mg.

X wt% MgH_2_ (x = 1, 5, and 10) was added together with Ni, TaF_5_, and VCl_3_ at the same time with a goal of preparing materials with better hydriding and dehydriding properties.

**Figure 3:**
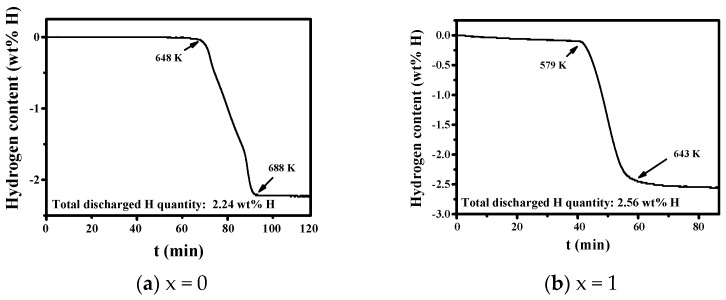

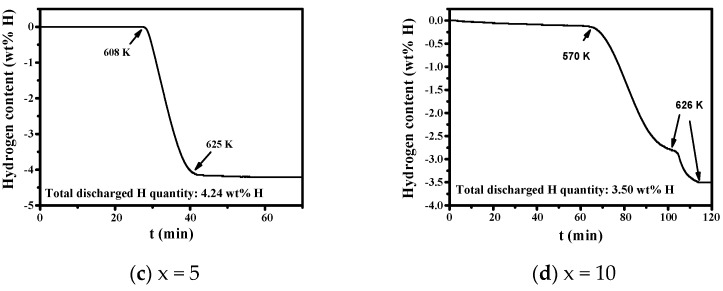
Desorbed hydrogen quantity versus time t curves for the as-milled Mg-xMgH_2_-1.25Ni-1.25TaF_5_-1.25VCl_3_; (**a**) x = 0, (**b**) x = 1, (**c**) x = 5, and (**d**) x = 10 when the sample was heated with a heating rate of 5–6 K/min.

Figure 3 shows the desorbed hydrogen quantity versus time t curves for the as-milled Mg-xMgH_2_-1.25Ni-1.25TaF_5_-1.25VCl_3_ (x = 0, 1, 5, and 10) when the sample was heated with a heating rate of 5–6 K/min. The ranges of temperature where hydrogen is released rapidly are 648–688 K, 579–643 K, 608–625 K, and 570–626 K, respectively. The addition of MgH_2_ lowers the temperature at which hydrogen begins to release rapidly. The curves of the samples with x = 0 and x = 10 show two stages. The total desorbed hydrogen quantities of the as-milled Mg-xMgH_2_-1.25Ni-1.25TaF_5_-1.25VCl_3_ (x = 0, 1, 5, and 10) were 2.24, 2.56, 4.24, and 3.50 wt% H, respectively.

**Figure 4:**
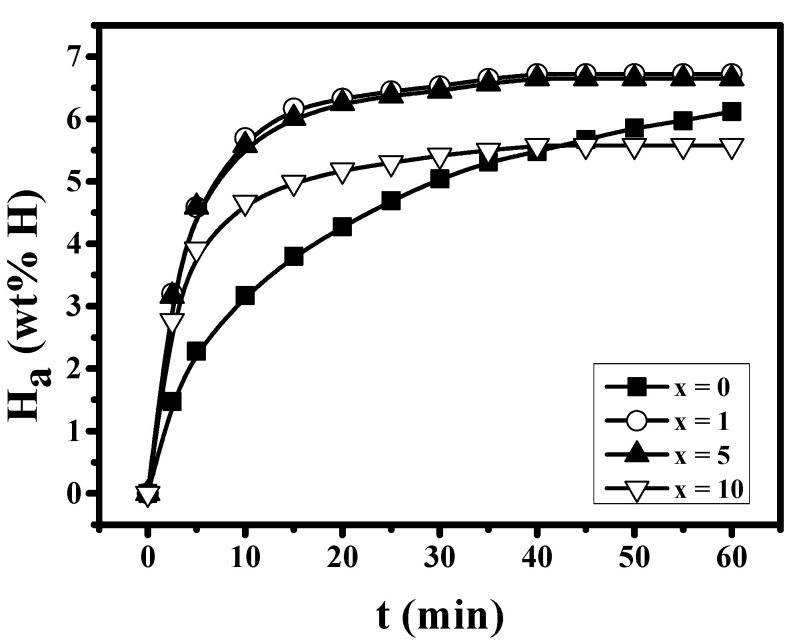
H_a_ versus t curves at 593 K under 12 bar H_2_ at n = 1 for Mg-xMgH_2_-1.25Ni-1.25TaF_5_-1.25VCl_3_ (x = 0, 1, 5, and 10).

The H_a_ versus t curves at 593 K under 12 bar H_2_ at n = 1 for Mg-xMgH_2_-1.25Ni-1.25TaF_5_-1.25VCl_3_ (x = 0, 1, 5, and 10) are shown in Figure 4. The sample with x = 0 has a quite high hydriding rate for the first 5 min, and the samples with x = 1, 5, and 10 have higher hydriding rates for the first 5 min than the sample with x = 0. The addition of MgH_2_ increases the hydriding rate for the first 5 min. The sample with x = 1 has the highest hydriding rate for the first 5 min, followed in order by the samples with x = 5, 10, and 0. The sample with x = 1 has the largest quantity of hydrogen absorbed for 60 min, H_a_ (60 min), followed in order by the samples with x = 5, 0, and 10. The addition of 1 and 5 wt% MgH_2_ increases H_a_ (60 min) to 6.72 wt% H (x = 1) and 6.65 wt% H (x = 5) from 6.12 wt% H (x = 0). Mg-1MgH_2_-1.25Ni-1.25TaF_5_-1.25VCl_3_ absorbs 3.20 wt% H for 2.5 min, 5.69 wt% H for 10 min, and 6.72 wt% H for 60 min. We define the effective hydrogen-storage capacity as the quantity of hydrogen absorbed for 60 min. The sample with x = 5 absorbs 5.58 wt% H for 10 min and has an effective hydrogen-storage capacity of 6.65 wt%.

Table 1 presents the variations of the H_a_ with time and hydriding rates for the first 5 min at 593 K under 12 bar H_2_ at n = 1 for Mg-xMgH_2_-1.25Ni-1.25TaF_5_-1.25VCl_3_ (x = 0, 1, 5, and 10).

The sample with x = 0 has quite a high hydriding rate for the first 5 min, and the samples with x = 1, 5, and 10 have higher hydriding rates for the first 5 min than the sample with x = 0. The addition of MgH_2_ increases the hydriding rate for the first 5 min. The samples with x = 1 and 5 have the highest hydriding rate for the first 5 min, followed in order by the samples with x = 10 and 0. The sample with x = 1 has the largest quantity of hydrogen absorbed for 60 min, H_a_ (60 min), followed in order by the samples with x = 5, 0, and 10. The addition of 1 and 5 wt% MgH_2_ increases H_a_ (60 min).

**Figure 5:**
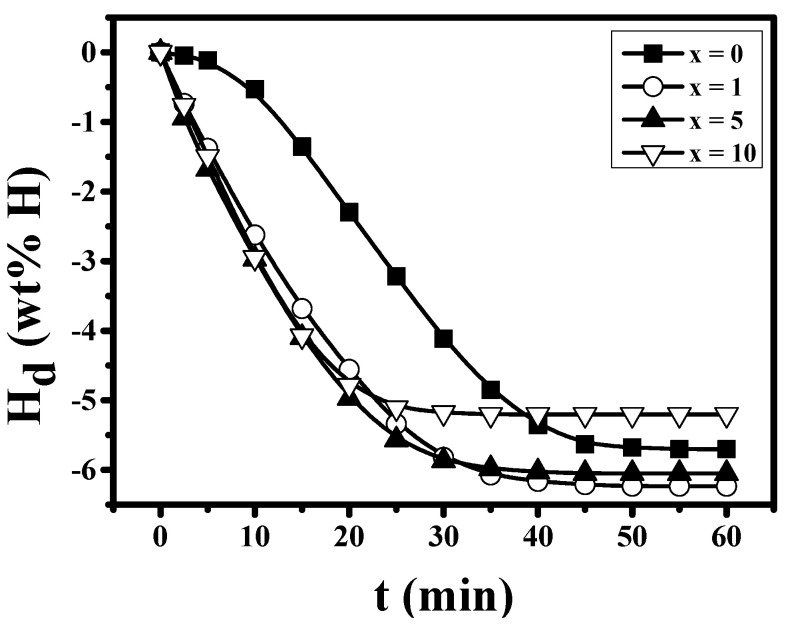
H_d_ versus t curves at 593 K under 1.0 bar H_2_ at n = 1 for Mg-xMgH_2_-1.25Ni-1.25TaF_5_-1.25VCl_3_ (x = 0, 1, 5, and 10).

The H_d_ versus t curves at 593 K under 1.0 bar H_2_ at n = 1 for Mg-xMgH_2_-1.25Ni-1.25TaF_5_-1.25VCl_3_ (x = 0, 1, 5, and 10) are shown in Figure 5. The sample with x = 0 exhibits an incubation period of about 2.5 min, and then the dehydriding rate increases gradually. After 15 min, the dehydriding rate of the sample with x = 0 is quite high and becomes very low after 45 min. Mg-xMgH_2_-1.25Ni-1.25TaF_5_-1.25VCl_3_ (x = 1, 5, and 10) have quite high dehydriding rates from the start. The addition of MgH_2_ increases the dehydriding rate for the first 5 min. The sample with x = 5 has the highest dehydriding rate for the first 5 min, followed in order by the samples with x = 10, 1, and 0. The sample with x = 1 has the largest quantity of hydrogen released for 60 min, H_d_ (60 min), followed in order by the samples with x = 5, 0, and 10. The addition of 1 and 5 wt% MgH_2_ increases H_d_ (60 min).

Table 2 presents the variations of H_d_ with time and the dehydriding rates for the first 5 min at 593 K under 1.0 bar H_2_ at n = 1 for Mg-xMgH_2_-1.25Ni-1.25TaF_5_-1.25VCl_3_ (x = 0, 1, 5, and 10).

The sample with x = 0 has a very low dehydriding rate for the first 5 min, and the samples with x = 1, 5, and 10 have much higher dehydriding rates for the first 5 min than the sample with x = 0. The sample with x = 5 has the highest dehydriding rate for the first 5 min, followed in order by the samples with x = 10, 1, and 0. The addition of MgH_2_ increases the dehydriding rate for the first 5 min. The sample with x = 1 has the largest quantity of hydrogen released for 60 min, H_d_ (60 min), followed in order by the samples with x = 5, 0, and 10. The addition of 1 and 5 wt% MgH_2_ increases H_d_ (60 min).

**Figure 6:**
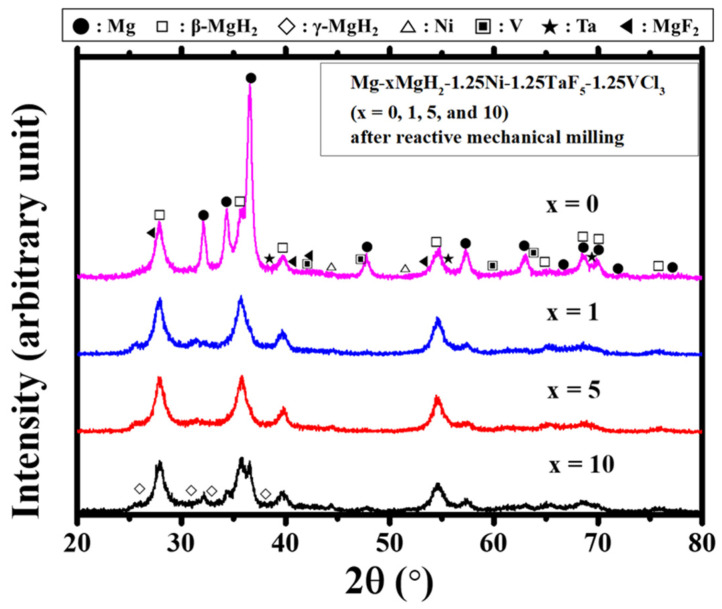
XRD patterns of Mg-xMgH_2_-1.25Ni-1.25TaF_5_-1.25VCl_3_ (x = 0, 1, 5, and 10) after reactive mechanical milling.

Figure 6 presents the XRD patterns of Mg-xMgH_2_-1.25Ni-1.25TaF_5_-1.25VCl_3_ (x = 0, 1, 5, and 10) after reactive mechanical milling. The samples contain Mg, β-MgH_2_, γ-MgH_2_, Ta, V, Ni, and MgF_2_. β-MgH_2_ and γ-MgH_2_ are formed by a reaction of Mg with hydrogen during reactive mechanical milling. Ta and MgF_2_ are formed due to a reaction of TaF_5_ with Mg. V is believed to be formed from a reaction of VCl_3_ with Mg. The reaction of VCl_3_ with Mg is reported to form MgCl_2_ together with V [32]. Ni remains unreacted after reactive mechanical milling. The intensity of the Mg peaks decrease in the samples containing MgH_2_. This is believed to be due to the decrease in Mg content in MgH_2_-added samples. Larger decrease in particle size due to reactive mechanical milling with the MgH_2_ mix is thought to have made the Mg peaks wider.

**Figure 7:**
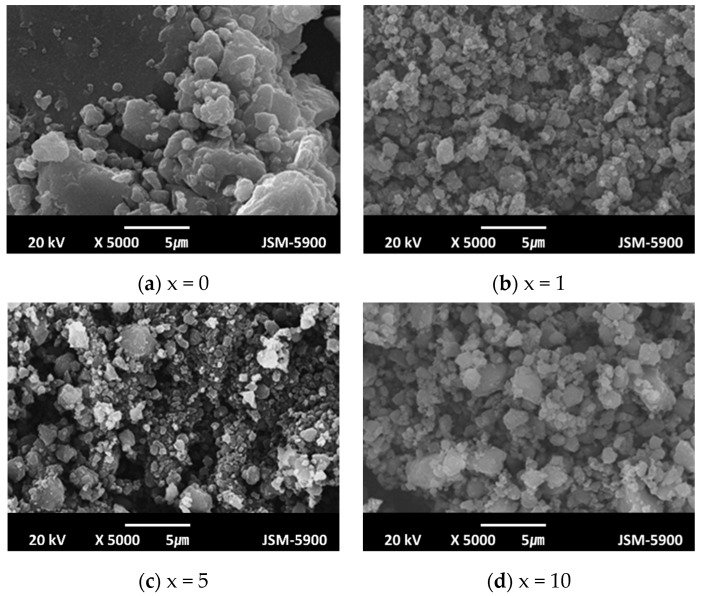
SEM micrographs of Mg-xMgH_2_-1.25Ni-1.25TaF_5_-1.25VCl_3_ (x = 1, 1, 5, and 10) after reactive mechanical milling; (**a**) x = 0, (**b**) x = 1, (**c**) x = 5, and (**d**) x = 10.

The SEM micrographs of Mg-xMgH_2_-1.25Ni-1.25TaF_5_-1.25VCl_3_ (x = 0, 1, 5, and 10) after reactive mechanical milling are presented in Figure 7. Particle sizes are not homogeneous, but Mg-xMgH_2_-1.25Ni-1.25TaF_5_-1.25VCl_3_ (x = 1 and 10) exhibit more homogeneous particle sizes than Mg-xMgH_2_-1.25Ni-1.25TaF_5_-1.25VCl_3_ (x = 0 and 5). The sample with x = 5 has the smallest particle size, followed in order by the samples with x = 1, 10, and 0. The samples with x = 5 and 1 have similar particle sizes. The sample with x = 0, not containing MgH_2_, has quite large particles, and the particles of this sample form agglomerates. The addition of MgH_2_ decreases the particle size.

**Figure 8:**
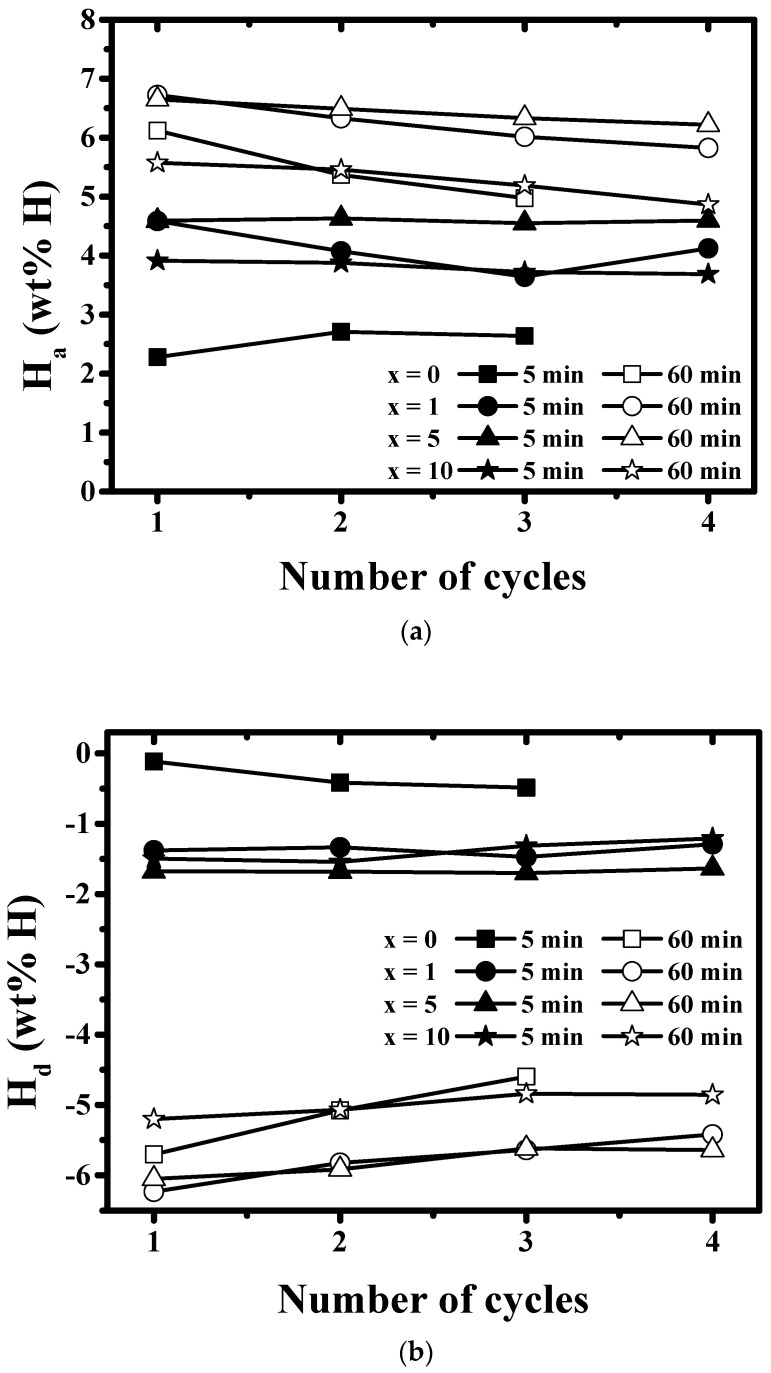
Variations of (**a**) H_a_ values for 5 min and 60 min at 593 K under 12 bar H_2_ and (**b**) H_d_ values for 5 min and 60 min at 593 K under 1.0 bar H_2_ with the number of cycles for Mg-xMgH_2_-1.25Ni-1.25TaF_5_-1.25VCl_3_ (x = 0, 1, 5, and 10).

The variations of H_a_ values for 5 min and 60 min at 593 K under 12 bar H_2_ with the number of cycles for Mg-xMgH_2_-1.25Ni-1.25TaF_5_-1.25VCl_3_ (x = 0, 1, 5, and 10) are shown in Figure 8a. The variations of H_d_ values for 5 min and 60 min at 593 K under 1.0 bar H_2_ with the number of cycles for Mg-xMgH_2_-1.25Ni-1.25TaF_5_-1.25VCl_3_ (x = 0, 1, 5, and 10) are shown in Figure 8b. The samples with x = 1, 5, and 10 have better hydriding and dehydriding cycling performance than the sample with x = 0, showing that the addition of MgH_2_ improves the cycling performance. The sample with x = 5 has the largest value of the quantity of hydrogen absorbed for 5 min, H_a_ (5 min), at each cycle and the best cycling performance. At n = 1, the sample with x = 1 has the largest value of the quantity of hydrogen absorbed for 60 min, H_a_ (60 min), and a slightly larger value of H_a_ (60 min) than the sample with x = 5. At n = 2−4, the sample with x = 5 has the largest values of H_a_ (60 min). However, the sample with x = 5 has the best cycling performance. The sample with x = 5 has the largest value of the quantity of hydrogen released for 5 min, H_d_ (5 min), at each cycle, and the best cycling performance. At n = 1, the sample with x = 1 has the largest value of the quantity of hydrogen released for 60 min, H_d_ (60 min), and a slightly larger value of H_d_ (60 min) than the sample with x = 5. However, the sample with x = 5 has the best cycling performance.

**Figure 9:**
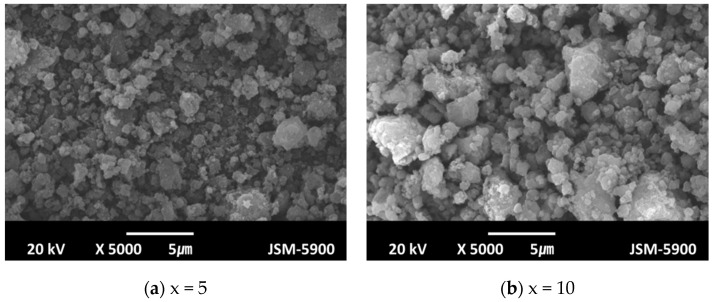
SEM micrographs of Mg-xMgH_2_-1.25Ni-1.25TaF_5_-1.25VCl_3_ (x = 5 and 10) after four hydriding and dehydriding cycles; (**a**) x = 5 and (**b**) x = 10.

Figure 9 shows the SEM micrographs of Mg-xMgH_2_-1.25Ni-1.25TaF_5_-1.25VCl_3_ (x = 5 and 10) after four hydriding and dehydriding cycles. Compared with the particles of these samples after reactive mechanical milling, those of the samples after four hydriding and dehydriding cycles are more agglomerated.

## 4. Discussion

The weight percentages of additives were relatively small (2.5 and 3.75 wt%) not to sacrifice the hydrogen-storage capacity of the samples. The addition of Ni together with TaF_5_ and VCl_3_ increases the quantity of hydrogen absorbed for 60 min of the TaF_5_ and VCl_3_-added Mg, as shown in Figure 1. Figure 2 shows that the addition of Ni together with TaF_5_ and VCl_3_ increases the maximum dehydriding rate and the quantity of hydrogen released for 60 min of the TaF_5_ and VCl_3_-added Mg.

Metallic hydrides are more brittle than their parent metals [33]. MgH_2_ is brittle [34]_._ The addition of MgH_2_ was expected to increase hydriding and dehydriding rates, as MgH_2_ may be pulverized during milling. To prepare materials with better hydriding and dehydriding properties, x wt% MgH_2_ (x = 1, 5, and 10) was added together with Ni, TaF_5_, and VCl_3_ at the same time.

Figure 3 shows that the as-milled Mg-xMgH_2_-1.25Ni-1.25TaF_5_-1.25VCl_3_ (x = 5) has the largest total desorbed hydrogen quantity of 4.24 wt%. Figure 4 shows that the samples with x = 1, 5, and 10 have higher hydriding rates for the first 5 min than the sample with x = 0. The samples with x = 1 and 5 have larger quantities of hydrogen absorbed for 60 min, H_a_ (60 min) than the sample with x = 0. Figure 5 shows that the samples with x = 1, 5, and 10 have higher dehydriding rates for the first 5 min than the sample with x = 0. The samples with x = 1 and 5 have larger quantities of hydrogen released for 60 min, H_d_ (60 min), than the sample with x = 0. These results show that the addition of MgH_2_ increases the hydriding and dehydriding rates for the first 5 min and the additions of 1 and 5 wt% MgH_2_ increase the quantity of hydrogen absorbed and released for 60 min, H_a_ (60 min) and H_d_ (60 min). This proves that the addition of MgH_2_ made the effects of reactive mechanical milling, which are explained in the following, stronger.

Figure 7 shows that after reactive mechanical milling, the sample with x = 5 has the smallest particle size, followed in order by the samples with x = 1, 10, and 0. Figure 7 also shows that the samples with x = 5 and 1 have similar particle sizes. A comparison of the results in Figure 4 and Figure 5 with the particle sizes in the SEM micrographs in Figure 7 shows that the smaller the particle size, the higher the hydriding rate for the first 5 min and the dehydriding rate for the first 5 min.

The reactive mechanical milling of Mg with Ni, TaF_5_, and VCl_3_ is thought to increase the hydriding and dehydriding rates by forming defects [35,36,37,38] (leading to easier nucleation) [39,40,41], making new clean surfaces (leading to an increase in the reactivity of Mg particles with hydrogen) [30,42], and decreasing the particle size of Mg (leading to a decrease in the diffusion distances of hydrogen atoms) [9,10,36,37,38,43]. Especially, the added Ni is believed to form the Mg_2_Ni phase, which has higher hydriding and dehydriding rates than Mg at the same conditions [36,44], contributing greatly to the increases in the reaction rates. The formed phases (β-MgH_2_, γ-MgH_2_, Ta, V, and MgCl_2_) are believed to help the reactive mechanical milling occur more effectively. The result that the addition of MgH_2_ increases the hydriding and dehydriding rates for the first 5 min and the result that the additions of 1 and 5 wt% MgH_2_ increase H_a_ (60 min) and H_d_ (60 min) show that the addition of MgH_2_ makes the effects of reactive mechanical milling stronger. Figure 8 shows that H_a_ (60 min) values decrease as n increases for all the samples. It is believed that the reason of this behavior is that particles coalesce more and more as n increases. The result that the addition of MgH_2_ improves the cycling performance, shown in Figure 8, indicates that the added MgH_2_ prevents particle from coalescing. The formed phases are also believed to help the sample have better cycling performance. After conducting many studies to improve the reaction kinetics of magnesium with hydrogen, researchers reported as the following. The dissociation rate of hydrogen molecules can be improved by adding catalytic metals, for example, Pd [45] and Co, Ni, or Fe [46]. Nucleation can be facilitated by creating active nucleation sites by mechanical treatment and/or alloying with additives [39]. The diffusion distance of hydrogen can be decreased by the mechanical treatment and/or alloying of Mg with additives, thereby reducing the magnesium particle size [43]. In addition, the hydrogen mobility can be improved by additives that create microscopic paths of hydrogen [43]. A rough surface of magnesium having many cracks and defects is thus considered more advantageous for hydrogen absorption [35].

Compared with the microstructures of these samples after reactive mechanical milling (shown in Figure 7), particles after four hydriding and dehydriding cycles are more agglomerated (shown in Figure 9). Agglomeration is believed to have led to the decreases in the absorbed and released hydrogen quantities of the samples.

Among Mg-xMgH_2_-1.25Ni-1.25TaF_5_-1.25VCl_3_ (x = 0, 1, 5, and 10), the samples with x = 1 and 5 have the highest hydriding rate for 5 min at n = 1, but the sample with x = 5 has the highest dehydriding rate for 5 min at n = 1. Among all the samples, the sample with x = 5 has the best cycling performance. The sample with x = 5 absorbs 5.58 wt% H for 10 min and has an effective hydrogen storage capacity of 6.65 wt%.

Table 3 presents the hydrogen-storage capacities of several Mg-based alloys. Conditions of sample preparation and reaction are given. The sample Mg-5MgH_2_-1.25Ni-1.25TaF_5_-1.25VCl_3_ has quite a high hydrogen-storage capacity. It has a higher hydrogen-storage capacity than Ni and Ti-added Mg alloys.

## 5. Conclusions

The addition of Ni together with TaF_5_ and VCl_3_ increased the quantity of hydrogen absorbed for 60 min and increased the maximum dehydriding rate and the quantity of hydrogen released for 60 min of the TaF_5_ and VCl_3_-added Mg. MgH_2_ was added together with Ni, TaF_5_, and VCl_3_, and Mg-x wt% MgH_2_-1.25 wt% Ni-1.25 wt% TaF_5_-1.25 wt% VCl_3_ (x = 0, 1, 5, and 10) were prepared by reactive mechanical milling. The addition of MgH_2_ increased the hydriding and dehydriding rates for the first 5 min, and the additions of 1 and 5 wt% MgH_2_ increased the quantities of hydrogen absorbed and released for 60 min, denoted H_a_ (60 min) and H_d_ (60 min), respectively. The comparison of H_d_ versus t and H_a_ versus t curves with SEM micrographs after reactive mechanical milling showed that the smaller the particle size, the higher the hydriding rate for the first 5 min, and the dehydriding rate for the first 5 min. The reactive mechanical milling of Mg with Ni, TaF_5_, and VCl_3_ is thought to increase the hydriding and dehydriding rates by forming defects, making new clean surfaces, and decreasing the particle size of Mg. The addition of MgH_2_ made the effects of reactive mechanical milling stronger. The formed phases (β-MgH_2_, γ-MgH_2_, Ta, V, and MgCl_2_) are also believed to have made these effects stronger. The addition of MgH_2_ improved cycling performance by preventing particles from coalescing. The formed phases are also believed to have helped the sample have the better cycling performance. Among Mg-xMgH_2_-1.25Ni-1.25TaF_5_-1.25VCl_3_ (x = 0, 1, 5, and 10), the sample with x = 5 had the highest hydriding and dehydriding rates for the first 5 min and the best cycling performance. The sample with x = 5 absorbed 5.58 wt% H for 10 min and had an effective hydrogen-storage capacity of 6.65 wt%.

## Figures and Tables

**Table 1:** Variations of the Ha with time and hydriding rates for the first 5 min at 593 K under 12 bar H_2_ at n = 1 for Mg-xMgH_2_-1.25Ni-1.25TaF_5_-1.25VCl_3_ (x = 0, 1, 5, and 10).

Mg-xMgH_2_-1.25Ni-1.25TaF_5_-1.25VCl_3_ at 593 K	Ha (wt% H)					Hydriding Rate for the First 5 min (wt% H/min)
	2.5 min	5 min	10 min	30 min	60 min	
x = 0	1.47	2.28	3.17	5.04	6.12	0.456
x = 1	3.2	4.59	5.69	6.52	6.72	0.917
x = 5	3.17	4.59	5.58	6.45	6.65	0.917
x = 10	2.77	3.91	4.66	5.42	5.57	0.783

**Table 2:** Variations of the H_d_ with time and the dehydriding rates for the first 5 min at 593 K under 1.0 bar H_2_ at n = 1 for Mg-xMgH_2_-1.25Ni-1.25TaF_5_-1.25VCl_3_ (x = 0, 1, 5, and 10).

Mg-xMgH_2_-1.25Ni-1.25TaF_5_-1.25VCl_3_ at 593 K	H_d_ (wt% H)					Dehydriding Rate for the First 5 min (wt% H/min)
	2.5 min	5 min	10 min	30 min	60 min	
x = 0	0.05	0.11	0.53	4.11	5.70	0.023
x = 1	0.74	1.38	2.62	5.82	6.24	0.276
x = 5	0.94	1.68	2.97	5.87	6.05	0.336
x = 10	0.76	1.50	2.95	5.18	5.20	0.299

**Table 3:** Hydrogen-storage capacities of several Mg-based alloys.

Composition	Ball Milling Time (h)	Reaction Temperature (K)	Reaction Hydrogen Pressure (Bar H_2_)	Hydrogen- Storage Capacity (wt% H)	Reference
Mg-2.5TaF_5_-2.5VCl_3_	6	593	12	5.86 (n = 1)	[30]
Mg-1.25TaF_5_-1.25VCl_3_	6	593	12	5.36 (n = 1)	this work
Mg-1.25Ni-1.25TaF_5_-1.25VCl_3_	6	593	12	6.12 (n = 1)	this work
Mg-5MgH_2_-1.25Ni-1.25TaF_5_-1.25VCl_3_	6	593	12	6.65 (n = 1)	this work
Mg-14Ni-6Ti	6	573	12	4.98	[27]
Mg-1.25Ni-1.25Ti	6	593	12	5.91	[47]

## References

[1] Von Colbe J.B., Ares J.-R., Barale J., Baricco M., Buckley C., Capurso G., Gallandat N., Grant D., Guzik M.N., Jacob I. (2019). Application of hydrides in hydrogen storage and compression: Achievements, outlook and perspectives. Int. J. Hydrog. Energy.

[2] Zhang X., Liu Y., Ren Z., Zhang X., Hu J., Huang Z., Lu Y., Gao M., Pan H. (2020). Realizing 6.7 wt% reversible storage of hydrogen at ambient temperature with non-confined ultrafine magnesium hydrides. Energy Environ. Sci..

[3] Liao W., Jiang W., Yang X.-S., Wang H., Ouyang L., Zhu M. (2020). Enhancing (de)hydrogenation kinetics properties of the Mg/MgH_2_ system by adding ANi5 (A = Ce, Nd, Pr, Sm, and Y) alloys via ball milling. J. Rare Earths.

[4] Pukazhselvan D., Sandhya K., Nasani N., Fagg D.P. (2021). Chemical transformation of additive phase in MgH_2_/CeO_2_ hydrogen storage system and its effect on catalytic performance. Appl. Surf. Sci..

[5] Paul D.R., Sharma A., Panchal P., Chaudhary S., Patidar D., Nehra S. (2020). Effect of ball milling and iron mixing on structural and morphological properties of magnesium for hydrogen storage application. Mater. Today Proc..

[6] Berezovets V., Denys R., Zavaliy I., Kosarchyn Y. (2021). Effect of Ti-based nanosized additives on the hydrogen storage properties of MgH_2_. Int. J. Hydrog. Energy.

[7] Yin Y., Li B., Yuan Z., Qi Y., Zhang Y. (2019). Enhanced hydrogen storage performance of Mg-Cu-Ni system catalyzed by CeO_2_ additive. J. Rare Earths.

[8] Liu P., Chen H., Yu H., Liu X., Jiang R., Li X., Zhou S. (2019). Oxygen vacancy in magnesium/cerium composite from ball milling for hydrogen storage improvement. Int. J. Hydrog. Energy.

[9] Song M.Y., Choi E., Kwak Y.J. (2020). Increase in the dehydrogenation rates and hydrogen-storage capacity of Mg-graphene composites by adding nickel via reactive ball milling. Mater. Res. Bull..

[10] Song M.Y., Lee S.H., Kwak Y.J. (2021). Improvement in the hydrogenation and dehydrogenation features of Mg by milling in hydrogen with vanadium chloride. Korean J. Met. Mater..

[11] Li Z., Liu X., Jiang L., Wang S. (2007). Characterization of Mg–20 wt% Ni–Y hydrogen storage composite prepared by reactive mechanical alloying. Int. J. Hydrog. Energy.

[12] Liang G., Huot J., Boily S., Van Neste A., Schulz R. (1999). Hydrogen storage properties of the mechanically milled MgH_2_–V nanocomposite. J. Alloy. Compd..

[13] Huot J., Pelletier J., Lurio L., Sutton M., Schulz R. (2003). Investigation of dehydrogenation mechanism of MgH_2_–Nb nanocomposites. J. Alloy. Compd..

[14] Fu Y., Groll M., Mertz R., Kulenovic R. (2008). Effect of LaNi_5_ and additional catalysts on hydrogen storage properties of Mg. J. Alloy. Compd..

[15] Vijay R., Sundaresan R., Maiya M., Murthy S.S., Fu Y., Klein H.-P., Groll M. (2004). Characterisation of Mg–x wt.% FeTi (x = 5–30) and Mg–40 wt.% FeTiMn hydrogen absorbing materials prepared by mechanical alloying. J. Alloy. Compd..

[16] Friedrichs O., Klassen T., Sanchez-Lopez J.C., Bormann R., Fernandez A. (2006). Hydrogen sorption improvement of nanocrystalline MgH_2_ by Nb_2_O_5_ nanoparticles. Scr. Mater..

[17] Gupta R., Agresti F., Russo S.L., Maddalena A., Palade P., Principi G. (2008). Structure and hydrogen storage properties of MgH_2_ catalysed with La_2_O_3_. J. Alloy. Compd..

[18] Zhang Y., Zhang W., Wei X., Yuan Z., Gao J., Guo S., Ren H. (2021). Catalytic effects of TiO_2_ on hydrogen storage thermodynamics and kinetics of the as-milled Mg-based alloy. Mater. Charact..

[19] Zhang Y., Wei X., Zhang W., Yuan Z., Gao J., Ren H. (2021). Catalytic effect comparison of TiO_2_ and La_2_O_3_ on hydrogen storage thermodynamics and kinetics of the as-milled La-Sm-Mg-Ni-based alloy. J. Magnes. Alloy.

[20] Zhang Y., Wei X., Zhang W., Yuan Z., Gao J., Qi Y., Ren H. (2020). Effect of milling duration on hydrogen storage thermodynamics and kinetics of Mg-based alloy. Int. J. Hydrog. Energy.

[21] Yong H., Wei X., Zhang K., Wang S., Zhao D., Hu J., Zhang Y. (2021). Characterization of microstructure, hydrogen storage kinetics and thermodynamics of ball-milled Mg_90_Y_1.5_Ce_1.5_Ni_7_ alloy. Int. J. Hydrog. Energy.

[22] Hou Z., Wei X., Zhang W., Yuan Z., Ge Q. (2021). Electrochemical hydrogen storage performances of Mg–Ti–Ni–Co–Al-based alloys prepared by mechanical milling. J. Phys. Chem. Solids.

[23] Wang W., Chen C., Chen L., Wang Q. (2002). Change in structure and hydrogen storage properties of La_2_Mg_16_Ni alloy after modification by mechanical grinding in tetrahydrofuran. J. Alloy. Compd..

[24] Sun D., Gingl F., Nakamura Y., Enoki H., Bououdina M., Akiba E. (2002). In situ X-ray diffraction study of hydrogen-induced phase decomposition in LaMg_12_ and La_2_Mg_17_. J. Alloy. Compd..

[25] Ouyang L., Dong H., Zhu M. (2007). Mg_3_Mm compound based hydrogen storage materials. J. Alloy. Compd..

[26] Yabe H., Kuji T. (2007). Thermal stability and hydrogen absorption/desorption properties of Mg_17_Al_12_ produced by bulk mechanical alloying. J. Alloy. Compd..

[27] Malka I., Pisarek M., Czujko T., Bystrzycki J. (2011). A study of the ZrF_4_, NbF_5_, TaF_5_, and TiCl_3_ influences on the MgH_2_ sorption properties. Int. J. Hydrog. Energy.

[28] Kumar S., Singh P.K., Rao G.N., Kojima Y., Kain V. (2018). Synergic effect of vanadium trichloride on the reversible hydrogen storage performance of the Mg MgH_2_ system. Int. J. Hydrog. Energy.

[29] Liu Y., Zou J., Zeng X., Wu X., Li D., Ding W. (2014). Hydrogen storage properties of a Mg–Ni nanocomposite coprecipitated from solution. J. Phys. Chem. C.

[30] Kwak Y.J., Song M.Y. (2018). Enhancement of the hydrogen uptake and release rates of Mg by the addition of TaF_5_ and VCl_3_ with reactive mechanical grinding. Nanosci. Nanotechnol. Lett..

[31] Song M.Y., Baek S.H., Bobet J.-L., Hong S.-H. (2010). Hydrogen storage properties of a Mg–Ni–Fe mixture prepared via planetary ball milling in a H_2_ atmosphere. Int. J. Hydrog. Energy.

[32] da Conceição M., Brum M., dos Santos D. (2014). The effect of V, VCl_3_ and VC catalysts on the MgH_2_ hydrogen sorption properties. J. Alloy. Compd..

[33] Mandzhukova T., Bobet J.-L., Khrussanova M., Peshev P. (2009). Hydrogen sorption properties of MgH_2_–NiCo_2_O_4_ composites activated mechanically under argon and hydrogen atmospheres. Mater. Res. Bull..

[34] Metallic hydrides. https://www.toppr.com/ask/content/story/amp/metallic-hydrides-9178/.

[35] Vigeholm B., Kjøller J., Larsen B., Pedersen A. (1983). Formation and decomposition of magnesium hydride. J. Less Common Met..

[36] Song M., Ivanov E., Darriet B., Pezat M., Hagenmuller P. (1987). Hydriding and dehydriding characteristics of mechanically alloyed mixtures Mg-x wt.% Ni (x = 5, 10, 25 and 55). J. Less Common Met..

[37] Song M.Y. (1995). Effects of mechanical alloying on the hydrogen storage characteristics of Mg-x wt% Ni (x = 0, 5, 10, 25 and 55) mixtures. Int. J. Hydrog. Energy.

[38] Song M.Y. (1995). Improvement in hydrogen storage characteristics of magnesium by mechanical alloying with nickel. J. Mater. Sci..

[39] Hjort P., Krozer A., Kasemo B. (1996). Hydrogen sorption kinetics in partly oxidized Mg films. J. Alloy. Compd..

[40] Song M.Y., Choi E., Kwak Y.J. (2019). Preparation of a Mg-based alloy with a high hydrogen-storage capacity by adding a polymer CMC via milling in a hydrogen atmosphere. Int. J. Hydrog. Energy.

[41] Song M.Y., Choi E., Kwak Y.J. (2018). Increase in the dehydrogenation rate of Mg–CMC (Carboxymethylcellulose, Sodium Salt) by adding Ni via hydride-forming milling. Met. Mater. Int..

[42] Song M.Y., Kwak Y.J. (2019). Effects of Zn(BH_4_)_2_, Ni, and/or Ti doping on the hydrogen-storage features of MgH_2_. Korean J. Met. Mater..

[43] Zaluska A., Zaluski L., Ström–Olsen J. (1999). Nanocrystalline magnesium for hydrogen storage. J. Alloy. Compd..

[44] Reilly J.J., Wiswall R.H. (1968). Reaction of hydrogen with alloys of magnesium and nickel and the formation of Mg_2_NiH_4_. Inorg. Chem..

[45] Krozer A., Kasemo B. (1989). Equilibrium hydrogen uptake and associated kinetics for the Mg-H_2_ system at low pressures. J. Phys. Condens. Matter.

[46] Stillesjö F., Ólafsson S., Hjörvarsson B., Karlsson E. (1993). Hydride formation in Mg/Ni-sandwiches studied by hydrogen profiling and volumetric measurements. Z. Phys. Chem..

[47] Song M.Y., Kwak Y.J. (2021). Enhancement of the hydrogenation and dehydrogenation characteristics of Mg by adding small amounts of nickel and titanium. Mater. Sci..

